# Impact of body composition on the prognosis of hepatocellular carcinoma patients treated with transarterial chemoembolization: A systematic review and meta-analysis

**DOI:** 10.1016/j.heliyon.2024.e25237

**Published:** 2024-02-02

**Authors:** Anrong Wang, Junfeng Li, Changfeng Li, Hui Zhang, Yingfang Fan, Kuansheng Ma, Qiang Wang

**Affiliations:** a Department of Vascular Surgery, The First Affiliated Hospital of Chongqing Medical University, Chongqing, China; b Department of Interventional Therapy, People's Hospital of Dianjiang County, Chongqing, China; c Department of Oncology, People's Hospital of Dianjiang County, Chongqing, China; d Institution of Hepatobiliary Surgery, Southwest Hospital, Army Medical University, Chongqing, China; e Department of Hepatobiliary Surgery, Third Affiliated Hospital, Southern Medical University, Guangzhou, China; f Division of Medical Imaging and Technology, Department of Clinical Science, Intervention and Technology (CLINTEC), Karolinska Institutet, Stockholm, Sweden; g Department of Radiology, Karolinska University Hospital Huddinge, Stockholm, Sweden

**Keywords:** Body composition, Sarcopenia, Transarterial chemoembolization, Hepatocellular carcinoma, Meta-analysis

## Abstract

**Objectives:**

To summarize current evidence about the influence of body composition on the prognosis of patients with hepatocellular carcinoma (HCC) after transarterial chemoembolization (TACE) treatment.

**Methods:**

Public databases were systematically searched to identify relevant studies published from the inception of the database up to May 2023. Studies that evaluated the association between body composition and clinical outcomes in HCC patients who underwent TACE were included. A pre-designed table was applied to summarize relevant information. Meta-analysis was performed to estimate the association of body composition with overall survival.

**Results:**

Fourteen studies were included in this review, including 3631 patients (sample size range: 56–908, median 186). All body composition measurements (including skeletal muscle area, visceral and subcutaneous adipose area, and bone mineral density) were based on computer tomography. The commonly used parameter was skeletal muscle index at 3rd lumbar vertebra level (8/14). Three studies evaluated the correlations of body composition changes with the prognosis after TACE. Most studies (12/14) identified body composition parameters as an independent indicator for overall survival, progression-free survival, and treatment response rate. The hazard ratio of different body composition parameters ranged from 1.01 to 2.88, and hazard ratio of body composition changes ranged from 1.88 to 5.93. The pooled hazard ratio of sarcopenia for overall survival was 1.38 (95 %CI: 1.20–1.58).

**Conclusions:**

Body composition seems to be an important prognostic factor for a poorer clinical outcome after TACE treatment in patients with hepatocellular carcinoma. Future prospective studies with a larger sample size are required to confirm these findings.

**Registration study:**

This study has been prospectively registered at the PROSPERO platform (https://www.crd.york.ac.uk/prospero/) with the registration No. CRD42022345602.

## 1 Introduction

Primary liver cancer ranks the fourth most common malignancy and the sixth leading cause of cancer-related death worldwide. [1] Hepatocellular carcinoma (HCC) is the main histopathological subtype of primary liver cancer (∼75 %). [1,2] Due to the latent characteristics of HCC, many patients have been in the advanced stage at first diagnosis, leaving patients ineligible for radical liver resection. [3] As a backbone treatment in interventional radiology, transarterial chemoembolization (TACE) has a fundamental role in the management of unresectable HCC [4,5], and has been regarded as a standard treatment option for patients with intermediate-stage HCC according to the recommendations from the Barcelona Clinic Liver Cancer (BCLC) staging system. [4,6] In real-world practice, TACE might be the most commonly used first-line treatment across all BCLC stages. [7] Furthermore, TACE is also frequently used as the first alternative treatment for patients with tumor recurrence after surgical resection or loco-regional ablation. [7,8].

However, the treatment response and outcomes after TACE treatment vary substantially given that the indications are heterogeneous and patients with BCLC stage B may have different tumor burdens, liver function, and physical status. [9,10] Several risk factors have been identified for a poor prognosis in patients undergoing TACE, including the alpha-fetoprotein level, tumor number and size, tumor thrombus, number of lobar involvements, Model of End-Stage Liver Disease score, and tumor imaging features (such as irregular margin and internal arteries). [9,11,12] Based on these risk factors, several clinical models have been proposed to predict TACE outcomes, such as the hepatoma arterial-embolization prognostic score [13], the “six-and-twelve” score [14], and the CITRUS-MICAN score. [15] Nevertheless, these models only focus on tumor and liver function while overlooking the impact of the systemic prognostic factor on outcomes of TACE treatment.

Among the prognostic risk factors, the impact of body composition (such as muscle mass and visceral adipose) on outcomes of patients with HCC has drawn increasing attention in recent years. [16,17] Sarcopenia, defined as progressive and generalized skeletal muscle loss with aging, is shown to be correlated with short overall survival (OS), higher postoperative complications, and poor immunotherapy response in patients with HCC. [[18], [19], [20]] Given that HCC often develops in the context of chronic liver disease and fibrosis/cirrhosis, and the treatment of TACE usually lasts for a long period, the incidence of sarcopenia may be higher in these patients. Many studies have explored the influence of body composition on the prognosis of HCC patients who underwent TACE, but the methodology and results varied. A summary of currently available studies is of importance for evidence-based patient management as it can provide a more robust understanding of their relationships, and outline the knowledge gaps in the field. Yet, such a summary remains lacking. This study, therefore, aimed to systematically summarize the approaches adopted for body composition analysis and their impact on the prognosis after TACE treatment in HCC patients.

## 2 Methods

The research protocol of this study was prospectively registered at the PROSPERO platform (https://www.crd.york.ac.uk/prospero/) with the registration number CRD42022345602. We present the following article in accordance with the Preferred Reporting Items for Systematic Reviews and Meta-Analyses reporting checklist [21], which is provided in Supplemental sTable 1. Checklist items (13, 14, 20, and 21) are not applicable for this review as no data synthesis involved in this study.

### 2.1 Literature search and research selection

Literature was systematically searched in four public databases: PubMed, Web of Science, Embase, and Cochrane Library. A search strategy combined Medical Subject Headings terms and free terms of the following keywords: “transarterial chemoembolization”, “body composition”,“sarcopenia”, “myopenia” and “osteopenia”. Literature search was performed on July 26, 2022 and last updated on May 15, 2023. Detailed literature search queries are provided in Supplemental sTable 2.

Studies satisfying the following criteria were included: 1) prospective or retrospective cohort or case-control studies; 2) patients with HCC who underwent TACE without treatment history (liver resection or radiofrequency ablation); 3) at least one parameter of body composition was applied; 4) outcomes included OS, progression-free survival, or the treatment response rate; 5) studies in the English language. Publications in the form of a conference abstract, narrative reviews, letters, and editorials were excluded. To reduce technical variability, studies involving bland transarterial embolization (TAE), hepatic arterial infusion chemotherapy, a mixture of TACE/TAE and hepatic arterial infusion chemotherapy were excluded. For the patient population that overlapped in two studies (detected through information of medical center, ethical approval number, study period and patient indications), the one with more detailed information on clinical outcomes was included. In addition, the previous review and the reference list of the retrieved studies were searched manually to detect any possible eligible research.

Study selection was performed by reading the title and abstract first. Potential eligible research was further confirmed by reviewing the full text. Two investigators (A.W & Q. W) conducted study search and selection independently, and the results were cross-validated. The disagreement was solved by consulting a senior researcher (K.M).

### 2.2 Data extraction and research quality assessment

A pre-designed table was used to extract the following information: basic information (first author, publish year, and country), study characteristics (study design, single/multiple center study, and sample size), study population characteristics (age, gender ratio, and indication), TACE procedure characteristics (treatment history, embolic agents used, chemotherapeutic drug and repeated TACE treatment), the definition of sarcopenia and parameters used, and clinical outcomes (OS, 1-, 5-year OS, treatment response rate, progression-free survival, and independent risk factors for OS) with hazards ratio and 95 % confidence interval (CI).

Research quality and risk of bias were assessed by using the Newcastle-Ottawa Scale tool, which assigns up to a maximum of 9 points to indicate the lowest risk of bias of a non-randomized study in three domains: selection of study groups (maximum of 4 points), comparability of groups (maximum of 2 points) and ascertainment of exposure/outcome (maximum of 3 points). The result of 7–9 points denotes high quality, 4–6 moderate quality, and 0–3 low quality. [22].

This step was conducted by the same two investigators (A.W & Q. W) independently. In case of disagreements, a third investigator (senior researcher K.M) was invited to adjudicate.

### 2.3 Statistical analysis and meta-analysis

The pooled hazards ratio (HR) with 95 %CI were computed to estimate the overall impact of body composition on overall survival after TACE. Heterogeneity among the studies was assessed through Cochrane's Q test and the calculation of *I*^2^. *I*^2^ values falling within the ranges of <25 %, 25 %–50 %, 50 %–75 %, and >75 % signified no, low, moderate, and high heterogeneity, respectively. The fixed-effects model would be employed if *I*^2^ < 50 % in the heterogeneity test, otherwise the random-effects model employed. A funnel plot was adopted to demonstrate publication bias. Subgroup analysis was not feasible in this study as the data extracted from the included studies were not categorized into distinct groups. A p value less than 0.05 was regarded as statistically significant. All the statistical analyses and meta-analysis were performed by using a package “meta” (version 6.5–0) in R program.

## 3 Results

**Fig. 1:**
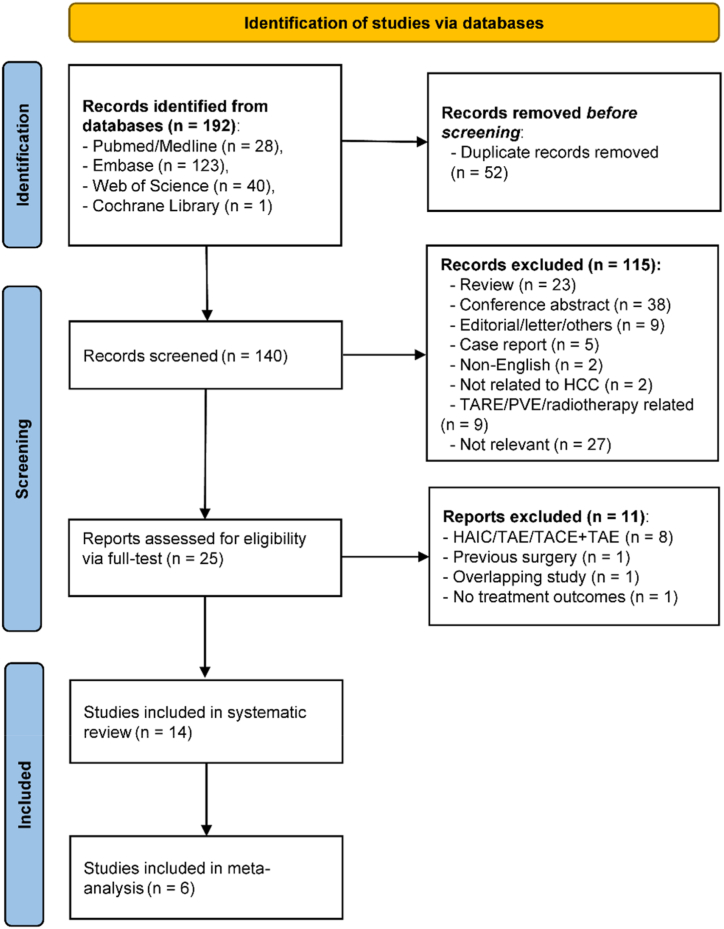
Process of study selection in this study. *Note: HAIC, hepatic arterial infusion chemotherapy; PVE, portal vein embolization; TACE, transarterial chemoembolization; TAE, transarterial embolization; TARE, transarterial radioembolization.*

The initial literature search yielded 192 records from the four databases. After removing 52 duplications, and 126 ineligible studies, 14 studies were eventually included in this review. [[23], [24], [25], [26], [27], [28], [29], [30], [31], [32], [33], [34], [35], [36]] The process of study selection is depicted in Fig. 1.

### 3.1 Study and patient characteristics

The 14 included studies were published between 2018 and 2023. All were retrospective except for one prospective study [27]; two were multi-center studies. [23,32] A total of 3631 patients were included, with a patient population ranging from 56 to 908 (median: 186). The average age ranged from 58 to 82; one study focused on octogenarians [24] and another on patients aged ≥65. [29] Except for two studies that assessed patients with HCC or liver metastases [26,34], all other studies focused exclusively on HCC. Most patients had a background of chronic hepatitis (hepatitis B and/or C virus (HBV/HCV) infection) (Table 1).

**Table 1:** Study and patient characteristics.

Study ID	Year of publication	Country/region	Study design	Study center	Sample size (cases)	Age (years)	Gender (M/F)	Indication	Chronic hepatitis (cases)	Treatment history	TACE type	Repeat TACE	NOS score
Parikh et al. [23]	2018	USA	RS	Two	165 (75 + 90)	60 vs 59^#^	73/2; 76/14^#^	HCC	HCV:63 (84 %)/55 (61 %)^#^	Treatment naïve	Unclear	Unclear	7
Cheng et al. [24]	2019	Japan	RS	Single	86	82	62/24	HCC (≥80 years)	HBV:4 (5 %); HCV:46 (53 %); HBV + HCV:9 (10 %)	Treatment naïve (62 %)	cTACE & DEB-TACE (91:9 %)	Yes	6
Fujita et al. [25]	2019	Japan	RS	Single	179	72	130/49	HCC	HBV:24 (13 %); HCV:85 (48 %)	Treatment naïve	cTACE & DEB-TACE	Yes	7
Loosen et al. [26]	2019	Germany	RS	Single	56	65	44/21	HCC (82 %) & liver metastases (18 %)	Unclear	Unclear	cTACE & DEB-TACE	Unclear	6
Hashida et al. [27]	2020	Japan	PS	Single	152	74 vs 75 †	98/54	HCC	HBV:12 (8 %); HCV:105 (69 %)	0-2 sessions of TACE	Unclear	Unclear	6
Li et al. [28]	2021	China	RS	Single	192	60	157/35	HCC (intermediate stage)	HBV:122 (64 %)	Treatment naïve	cTACE	Yes (every 6–8 weeks)	8
Lim et al. [29]	2021	Korea	RS	Single	266	70	187/79	HCC (≥65 years)	HBV:155 (58 %); HCV:60 (23 %)	Treatment naïve	cTACE & DEB-TACE	Unclear	8
Zheng et al. [30]	2021	China	RS	Single	75	≥50 (63 %)	63/12	HCC	Unclear	Treatment naïve	cTACE	Yes (1 month)	6
Zhang et al. [31]	2022	China	RS	Single	228	59	175/53	HCC	HBV:194 (85 %)	Treatment naïve	cTACE	Yes	9
Muller et al. [32]	2022	Germany	RS	Multiple	908	67	732/176	HCC	Viral hepatitis: 270 (30 %)	Treatment naive	Unclear	Unclear	7
Chien et al. [33]	2022	Taiwan	RS	Single	260	64	192/68	HCC	HBV:141 (54 %); HCV:110 (42 %)	Treatment naive	cTACE	Yes (every 2–3 months)	9
Loosen et al-2 [34]	2022	Germany	RS	Single	89	69	61/28	HCC & liver metastasis	HBV:14 %; HCV:27 %	Unclear	cTACE & DEB-TACE	Unclear	6
Bannangkoon et al. [35]	2023	Thailand	RS	Single	611	61	445/166	HCC	HBV:301 (49 %); HCV:135 (22 %); HBV + HCV:7 (1 %)	Treatment naive	cTACE	Unclear	8
Wang et al. [36]	2023	China	RS	Single	364	58	300/64	HCC	HBV:247 (68 %)	Treatment naive	cTACE	Yes (every 6–8 weeks)	8

Note: # Center 1 vs. Center 2; †control group vs. cancer rehabilitation group; cTACE, conventional TACE; DEB-TACE, drug-eluting bead TACE; HBV, hepatitis B virus; HCC, hepatocellular carcinoma; HCV, hepatitis C virus; NOS, Newcastle-Ottawa Scale; PS, prospective study; RS, retrospective study; TACE, transarterial chemoembolization.

One study evaluated patients with TACE treatment history [27], two studies did not describe such information [26,34], and the others evaluated patients with TACE treatment-naïve (11/14). Five studies applied techniques of both conventional TACE and drug-eluting bead TACE [[24], [25], [26],^29^,^34^], six studies focused on conventional TACE [28,30,31,33,35,36], while specific techniques were not listed in three studies. [23,27,32] Seven studies reported that repeated TACE was carried out once viable residual tumors were detected during follow-up examinations. [24,25,28,30,31,33,36] Detailed information is shown in Table 1.

Most of the included studies were moderate-to-high quality with median Newcastle-Ottawa Scale score of 7 (range: 6–9) (Table 1).

### 3.2 Body composition measurement

All measurements in the 14 studies were based on computed tomography (CT). The commonly used parameter was skeletal muscle index (SMI) at the third lumbar vertebra level (8/14), followed by psoas muscle index (4/14) and bone mineral density (3/14). A composite parameter combining skeletal muscle mass and visceral adipose was applied in one study (muscle depletion with visceral adiposity, MDVA). [29] Three studies evaluated the changes in body composition before and after TACE treatment. [25,27,30] Muscle function (grip strength, 10-m walking speed, and 6-min walking test) was measured in one study. [27] One study adopted analytic morphomics [23], which is a novel approach that uses high-throughput semi-automated image-processing techniques to assess body composition. [37] The measurement of these parameters, their corresponding definitions and cut-off value used are summarized in Table 2.

**Table 2:** Body composition measurement.

Study ID	Imaging technique	Body composition measurement	Parameters	Cut-off value
Parikh et al. [23]	CT	Analytic morphomics at 11th thoracic vertebra (T11)	VAT, dorsal muscle area etc. (12 parameters in total)	56th percentile of VAT
Cheng *et al*. [24]	CT	Skeletal muscle area at third lumbar vertebra (L3)	SMI	SMI ≤41 cm^2^/m^2^ for women and ≤53 cm^2^/m^2^ for men with body mass index ≥25, and ≤43 cm^2^/m^2^ for men and women with body mass index <25
Fujita et al. [25]	CT	Major × minor axis of the psoas muscle at L3; Its change per month during the TACE period	PMI, ΔPMI	PMI <6.0 cm^2^/m^2^ for men and <3.4 cm^2^/m^2^ for women
Loosen et al. [26]	CT	Sum of the longest and the perpendicular diameter of psoas muscle between L3 and L4	PMI	PMI <13.39 mm/m^2^
Hashida et al. [27]	CT	Skeletal muscle area, VFA at the umbilical level; Changes of skeletal muscle area and VFA, grip strength, 10-m walking speed, and 6-min walking test before and after TACE	ΔSMI, ΔVFA; Δgrip strength; Δ10 m walking speed; Δ6-min walk test	NA
Li et al. [28]	CT	Skeletal muscle area, VFA and SFA at L3	SMI, Muscle density, VAT index, VAT, SAT index, SAT density	Median of VAT
Lim et al. [29]	CT	Skeletal muscle area, VFA and SFA at L3	SMI, VFA/SFA ratio (Muscle depletion with visceral adiposity)	Below the median SMI and above the median VFA/SFA ratio value sex-specifically
Zheng et al. [30]	CT	CSAm, SFA and VFA at L3; BMD of mean of L1 and L2 Change of CSAm, SFA, VFA and BMD between pre and after TACE (120 days)	ΔCSAm, ΔSFA, ΔVFA, ΔBMD	NA.
Zhang et al. [31]	CT	Sum of the longest and the perpendicular diameter of psoas muscle at L3; Skeletal muscle area at L3	PMI, SMI	PMI: 42.28 mm/m^2^ (men),37.42 mm/m^2^ (women); SMI: 45.95 cm^2^/m^2^ (men), 33.96 cm^2^/m^2^ (women)
*Muller* et al. [32]	CT	BMD at T11	BMD	114 HU
*Chien* et al. [33]	CT	Psoas muscle area at L3	PMI	6.36 cm2/m2 (men), 3.92 cm2/m2 (women)
*Loosen* et al.*-2* [34]	CT	Skeletal muscle area, skeletal muscle density, BMD, VFA, SFA at L3	SMI, Median muscle density, BMD,VFA,SFA	SMI: 44.43 & 37.76 cm2/m2; median muscle density: 34.5 HU; BMD:159.5 HU; VFA: 401.4 cm2; SFA:180.4 cm2.
*Bannangkoon* et al. [35]	CT	Skeletal muscle density and area at L3	SMD, SMI	Myosteatosis: SMD: 44.4 HU (men), 39.3 HU (women); Sarcopenia: SMI:36.2 cm2/m2 (men), 29.6 cm2/m2 (women);
*Wang* et al. [36]	CT	Skeletal muscle area at L3	SMI	49 cm2/m2 (men); 36 cm2/m2 (women)

Note: BMD, bone mineral density; CSAm, cross-sectional area of paraspinal muscles; CT, computed tomography; HU, Hounsfield unit; NA, not available/applicable; PMI, psoas muscle index; SFA, subcutaneous fat area; SMI, Skeletal muscle index; TACE, transarterial chemoembolization; VFA, visceral fat area; VAT, visceral adipose tissue density; ΔCSAm, change of CSAm; ΔBMD, change of BMD; ΔPMI, change of PMI; ΔSMI, change of SMI; ΔSFA, change of SFA; ΔVFA, change of VFA.

Detailed information about differences between chronic hepatitis group and its counterpart group, or between different etiology groups can be found in Supplemental sTable 3. Interestingly, among six studies with available data, most of them (5/6) showed that there was not a significant difference in body composition between HBV/HCV group and non-HBV/HCV groups.

### 3.3 Prognosis of TACE treatment

#### 3.3.1 Overall survival (OS)

Eight studies compared the OS difference between different body composition groups, and all studies showed a significant difference with an OS of 9.4–18.0 months and 14.9–43.0 months for low and high body composition groups respectively (three studies claimed a significant result but not provide specific figures [25,30,34]). Interestingly, one study did not identify any significant body composition parameter but the performance status associating with OS. [24].

Among three studies that evaluated the correlation between body composition changes and OS, a significant result was observed in two studies [25,30]; the other study revealed that the difference in OS was not significant between the decrease and increase of SMI groups (459 vs. 526 days, p = 0.62) [27]. However, the latter study detected a significant difference between cancer rehabilitation and control groups for OS (529 vs. 369 days, p = 0.03) [27].

#### 3.3.2 One-year survival

One-year survival rate was assessed in four studies: three studies identified a significant difference of one-year survival rate between different body composition groups [23,29,34] (one study did not shown the specific figure [34]), one study demenstrated a one-year survival rate of 59 % for myosteatosis group (compared with 74 % in non-myosteatosis group), 60 % for sarcopenia group (71 % for non-sarcopenia group) and statistical analysis was not performed [35].

#### 3.3.3 Five-year survival

One study detected a significant difference between MDVA and non-MDVA groups in five-year survival (30.4 % vs. 49.7 %) [29]. In subgroup analysis, these results were consistent with Child-Pugh A and Child-Pugh B groups. The other study showed a five-year survival rate of 6 % vs 15 % in myosteatosis vs. non-myosteatosis groups and 10 % vs 12 % in sarcopenia vs non-sarcopenia groups [35].

#### 3.3.4 Progression-free survival

One study reported that the progression-free survival was significantly different between VAT and non-VAT groups (6.6 vs. 15.4 months) [28].

#### 3.3.5 Treatment response rate

Four studies evaluated the relationship between body composition and TACE treatment response: one study detected a significant difference in treatment response rate between MDVA and non-MDVA groups [29], one study found that myosteatosis, rather than sarcopenia, significantly affected the treatment response rate [35] and there was not any significant difference between different body composition groups in two studies [26,34].

### 3.4 Risk factors for OS after TACE

#### 3.4.1 Body composition parameters

**Fig. 2:**
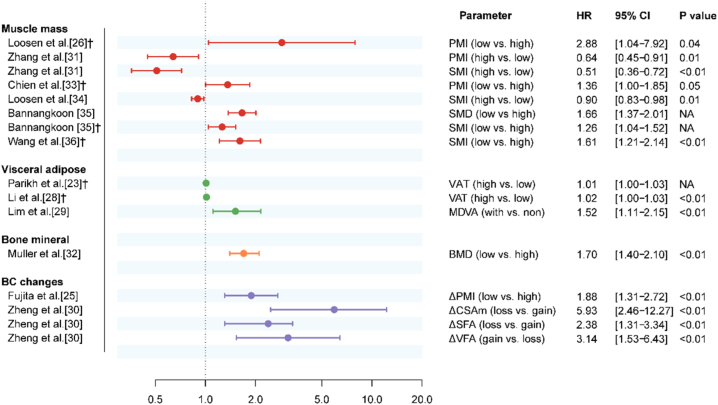
Forest plot illustrates the hazard ratio (HR) of body composition parameters for overall survival at multivariable regression analysis. *Note: † studies included in meta-analysis. BC, body composition, BMD, bone mineral density, CI, confidence interval, PMI, psoas muscle index, SMD, skeletal muscle density, SMI, skeletal muscle index; MDVA, muscle depletion with visceral adiposity; NA, not available; VAT, visceral adipose tissue density; ΔCSAm, change of the cross-sectional area of paraspinal muscles; ΔPMI, change of PMI; ΔSFA, change of subcutaneous fat area; ΔVFA, change of visceral fat area.*

The hazard ratio (HR) of different body composition parameters ranged from 1.01 to 2.88 [excluding two studies that set sarcopenia as the reference level [31,34]]. Regarding the longitudinal changes in body composition, Fujita et al. reported a significant ΔPsoas muscle index (HR:1.88) as a predictive factor for OS [25]. In another study, three parameters (ΔCSAmuscle, ΔSFA, ΔVFA) were significant predictive factors, with HR ranging from 2.38 to 5.93 [30]. The results of these parameters at multivariable Cox regression analysis for OS are depicted in Fig. 2.

**Fig. 3:**
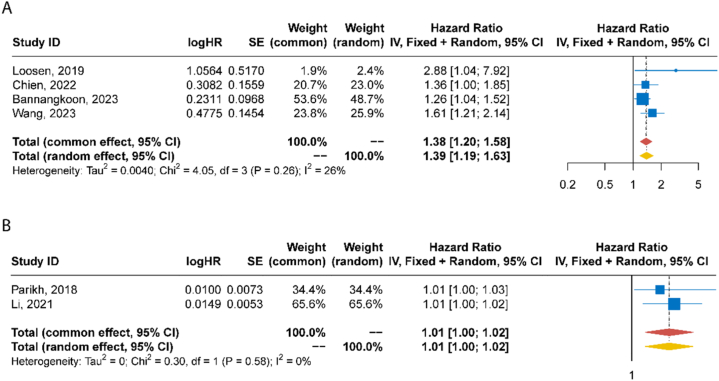
Forest plot showing the hazard ratio for overall survival after TACE between sarcopenia (low SMI or PMI) and non-sarcopenia (high SMI or PMI) (A), between high and low VAT (B). *Note: CI, confidence interval; HR, hazard ratio; PMI, psoas muscle index; SE, standard error, SMI, skeletal muscle index, VAT, visceral adipose tissue density*.

**Fig. 4:**
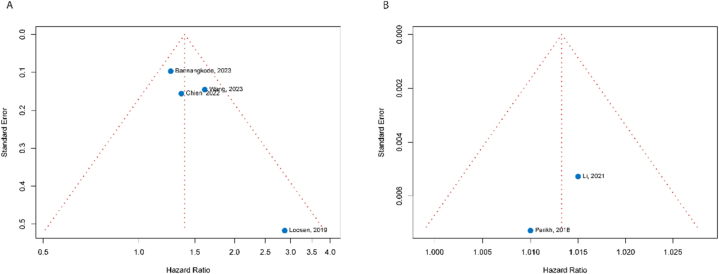
Funnel plots of hazard ratio of sarcopenia for overall survival in four studies (A), of visceral adipose tissue tensity for overall survival in two studies (B).

Six studies were included in meta-analysis, in which four involved muscle mass [26,33,35,36] and two visceral adiposity [23,28]. Meta-analysis results showed that the pooled HR of sarcopenia (defined by low SMI or PMI) for OS after TACE in HCC patients was 1.38 (95 %CI: 1.20–1.58). The *I*^2^ was 28 %, indicating a low heterogeneity among the four studies (Fig. 3A). The pooled HR of high VAT for OS was 1.01 (95 %CI:1.00–1.02) with *I*^2^ of 0 % (Fig. 3B). Fig. 4A and B demonstrates funnel plots of the HRs for the relationship between sarcopenia and OS, between VAT and OS after TACE, respectively. Due to limited number of studies, statistical test was not performed to detect the potential publication bias.

#### 3.4.2 Clinical variables

Independent clinical prognostic variables s associated with OS and detected at least two studies were: albumin level (5/12), Child-Pugh grade (4/12), alpha-fetoprotein level (4/12), tumor size (4/12), tumor number (4/12), the Tumor, Node, Metastasis stage (2/12), BCLC stage (2/12), mRECIST (2/12), bilirubin level (2/12) and portal vein thrombosis (2/12). Detailed information is provided in Table 3.

**Table 3:** Outcomes between different body composition groups and the independent indicators for overall survival.

Study ID	Groups	Overall survival	1-year survival	5-year survival	Progress free survival	Treatment response	Independent body composition indicator for overall survival	Independent clinical indicators for overall survival
Parikh et al. [23]	High vs. low VAT	NA	39 % vs 78 %* (Center 1); 44 % vs 72 %* (Center 2)	NA	NA	NA	VAT	Child-Pugh class B, TNM stage III or IV
Cheng et al. [24]	Performance status ≥1 vs. 0	29.3 vs. 43.2 months*	NA	NA	NA	NA	No	Performance status
Fujita et al. [25]	Low vs. high change of PMI	Significant (data not shown)	NA	NA	NA	NA	ΔPMI	AFP,mRECIST
Loosen et al. [26]	Low vs high PMI	16.4 vs 43.0 months*	NA	NA	NA	N.S (data not shown)	PMI	Albumin
Hashida et al. [27]	Decrease vs. increase of SMI	459 vs. 526 days (N.S); 369 vs. 529 days* (control vs. cancer rehabilitation)	NA	NA	NA	NA	No	Cancer rehabilitation, Child-Pugh class; Number of TACE sessions at baseline
Li et al. [28]	High vs. low VAT	17.6 vs 25.1 months*	NA	NA	6.6 vs. 15.4 months*	NA	VAT	No
Lim et al. [29]	MDVA vs non-MDVA	NA	83.5 % vs 91.4 %* 85.7 % vs 91.1 % *(BCLC-A) 80.0 % vs 90.9 %*(BCLC-B)	30.4 % vs 49.7 %*; 28.6 % vs 49.5 %*(BCLC-A); 42.4 % vs 25.7 %*(BCLC-B at 6 years)	NA	82.3 % vs 75.9 %* (Objective response rate)	MDVA	Age, MELD score, tumor size, serum albumin, platelet count, BCLC stage, not obtaining an objective response
Zheng et al. [30]	Gain vs loss of CSAm, SFA, VFA	Significant (data not available)	NA	NA	NA	NA	ΔCSAm, ΔSFA, ΔVFA	Child-Pugh class, portal vein thrombosis
Zhang et al. [31]	Low vs high PMI/SMI	16.9 vs 38.5 months*(PMI); 15.8 vs 33.5 months*(SMI)	NA	NA	NA	NA	PMI/SMI	AFP, Child-Pugh class, Tumor number, Metastases, BCLC stage C
Muller et al. [32]	Low vs high BMD	16.2 vs 22.2 months*	NA	NA	NA	NA	BMD	Albumin, bilirubin, tumor size, tumor number
Chien et al. [33]	Sarcopenia vs non-sarcopenia	18 vs 25 months*	NA	NA	NA	NA	Sarcopenia (PMI)	Tumor size, tumor number, AFP, albumin, major venous thrombosis
Loosen et al-2 [34]	Low vs high body composition parameters	Significant for SMI (data not shown)	Significant for SMI (data not shown)	NA	NA	NS for all body composition parameters	SMI	Lactate dehydrogenase
Bannangkoon et al. [35]	Myosteatosis vs non-myosteatosis; Sarcopenia vs non-sarcopenia	15.9 vs 27.1 months*; 16.6 vs 23.8 months*	59 % vs 74 %; 60 % vs 71 %	6 % vs 15 %; 10 % vs 12 %	NA	Significant for myosteatosis; NS for sarcopenia	Myoseatosis (SMD), sarcopenia (SMI)	No
Wang et al. [36]	Sarcopenia vs non-sarcopenia	9.4 vs 16.2 months* (training cohort); 10.6 vs 14.9 months*(validation cohort)	NA	NA	NA	NA	Sarcopenia (SMI)	AFP level ≥200 ng/mL, neutrophil-to-lymphocyte ratio ≥4.0, albumin-bilirubin grade, tumor size (≥5 cm), and tumor number (≥2)

Note: * statistically significant (p < 0.05); AFP, alpha-fetoprotein; BCLC, the Barcelona Clinic Liver Cancer (BCLC) staging system; CSAm, cross-sectional area of paraspinal muscles; MELD, Model of End-Stage Liver Disease scoring system; MDVA, Muscle depletion with visceral adiposity; mRECIST, modified Response Evaluation Criteria in Solid Tumors; NA, not available/applicable; NS, not significant; PMI, psoas muscle index; SMI, skeletal muscle index; TACE, transarterial chemoembolization; TNM, tumor, node, metastasis staging system; VAT, visceral adipose tissue density; ΔCSAm, change of the cross-sectional area of paraspinal muscles; ΔPMI, change of PMI; ΔSFA, change of subcutaneous fat area; ΔVFA, change of visceral fat area.

## 4 Discussion

This study systematically reviewed the association between body composition and the clinical outcomes after TACE treatment in patients with HCC. The results showed that the body composition parameters (including skeletal muscle mass, visceral fat, and bone mineral density) are an independent prognostic factor for OS, progression-free survival, and treatment response rate after TACE treatment. To the best of our knowledge, this is the first systematic review that comprehensively summarizes current evidence on this topic. These findings highlight the role of body composition parameters as prognostic indicators for HCC patients treated with TACE, which can potentially lead to the development of novel prognostic models for tailoring treatment strategies and optimizing patient care. Meanwhile, the limitations detected in this study warrant further investigation and will shape future research directions.

All reviewed studies used CT for body composition measurement. It is reasonable as CT examination is the most commonly used imaging modality in clinical practice not only for detection, diagnosis and staging of HCC but also for monitor and follow-up after treatment. Currently, several modalities are available for body composition analysis, such as dual-energy X-ray absorptiometry, and bioelectrical impedance analysis [38]. These methods are more widely available and easy-to-use. However, they involve additional exam and costs [32]. Furthermore, they cannot provide the distribution information, which is of clinical importance to evaluate ectopic fat, for example liver steatosis, or myosteatosis [39]. In the contrary, CT and magnetic resonance imaging (MRI) are clinical routinely-used imaging modalities. Although both CT and MRI are recognized as the gold standard approach for body composition measurement due to their high accuracy and reproducibility [40], the wide application of MRI is hampered by its relatively long exam time, high equipment cost, low accessibility and various exam contraindications. Besides, MRI is also limited by providing the information of bone mineral density and vascular calcium load. Therefore, CT is increasingly applied for comprehensive body composition analysis [39].

The parameters adopted in the reviewed studies included skeletal muscle mass, visceral adiposity, their changes before and after TACE treatment, and bone mineral density. For skeletal muscle mass measurement, SMI, which is calculated by dividing the cross-sectional area of skeletal muscle at a specific anatomical site (usually the level of the third lumbar vertebra) by the height squared (cm^2^/m^2^), is the widely used parameter in body composition-related research [41], The diagnosis of sarcopenia is mainly based on SMI. Lower SMI values are associated with increased frailty, poor physical function, and worse clinical outcomes in various disease conditions, including cancer, and liver disease [42]. Most studies (8/14) adopted this parameter in this review However, the cutoff value of SMI for sarcopenia definition varies across different studies [18].

Psoas muscle index (PMI), which is derived from the longest diameter and perpendicular diameter of the psoas muscle at the third level of the lumbar vertebra, is often used as a proxy for overall skeletal muscle mass, and its assessment provides insights into muscle wasting, particularly in the context of cancer and surgery [43]. PMI was detected as an independent indicator for OS in around one-third of the included studies [25,26,31,33]. One included study compared SMI and PMI in predicting the overall survival after TACE and showed that the predictive ability of PMI was comparable to that of SMI [31]. Compared with SMI, the measurement of PMI is simpler, takes less time, and does not require special software. Under this context, PMI may serve as a promising alternative for SMI and has the potential to be easily implemented in clinical practice.

Visceral adipose tissue refers to the measurement of the amount of fat accumulated within the abdominal cavity around the internal organs, such as the liver, or pancreas. Previous studies have shown that visceral adipose tissue is positively correlated with biomarkers of inflammation, metabolic disturbances and angiogenesis [44], which may play a fundamental role in cancer development and progress. Visceral adipose tissue has been revealed to be associated with the risk and prognosis of several tumors, including HCC [45]. In four included studies that simultaneously evaluated both skeletal muscle and visceral adipose compartments, two detected only visceral adipose-related parameters predictive for OS [23,28]. The difference between these two body composition compartments in correlation with patient prognosis after TACE requires more studies. On the other hand, the role of a combination of sarcopenia and obesity (namely “sarcopenic obesity”) in therapeutic outcomes of HCC has drawn increasing attention over recent years [46]. A meta-analysis that evaluated the impact of sarcopenic obesity on treatment outcomes in gastrointestinal surgical oncology showed that sarcopenic obesity was predictive of poor overall survival and disease-free survival after gastrointestinal surgery, with a hazard ratio of 1.73 and 1.41, respectively [47]. In the current review, one study adopted a composite parameter (MDVA, a concept similar to sarcopenic obesity) to improve the predictive accuracy for OS [29]. Similar to that meta-analysis, MDVA yielded an HR of 1.5 for OS (p = 0.009) [29].

Given that TACE treatment often involves a relatively long period, body composition may experience changes during this time. The dynamic changes in body composition may contain useful information for patient outcome prediction. Two included studies confirmed that muscle and fat mass changes were significantly correlated with overall survival after TACE treatment. Their HR values ranged from 1.8 to 5.9 (median: 2.4) [25,30]. On the other hand, body composition is modifiable, and patients can be guided to do aerobic endurance exercise and muscle training [48] or are given diet and nutritional support to improve outcomes after surgery [49]. Protein-enriched diets or branched-chain amino acids supplementation have been shown to improve sarcopenia-related indices, such as SMI [50,51]. In this review, one study compared the survival difference between cancer rehabilitation with control groups, and the former group had a significantly higher survival rate [27]. Interestingly, this study did not identify any significant indicator among the parameters of the muscle and visceral fat changes for overall survival prediction [27].

As emphasized by the European Working Group on Sarcopenia in Older People 2, assessment of muscle function (including strength and performance) has an equivalent role to muscle mass assessment in diagnosing sarcopenia [52]. Poorer muscle strength and physical performance are usually associated with a physical disability, prolonged hospital stays, poor quality of life, and higher adverse events in older people [53]. Endo et al. showed that grip strength, rather than SMI, was an independent factor for a poor prognosis of unresectable HCC patients in a small sample size [54]. Among the 14 included studies, only one evaluated muscle function by calculating the changes in grip strength, 10-m walking speed, and 6-min walk test before and after TACE therapy [27]. However, in that study, none of these parameters were significantly correlated with the survival rate. Future studies are warranted to compare the difference between muscle mass and muscle function and their changes in predicting patient prognosis.

Although most studies detected the body composition parameters as an independent indicator for a poorer clinical outcome after TACE treatment, their hazard ratio was relatively low (median: 2.4), and the lower bound of the confidence interval in three studies approximately equaled to 1 [23,26,28], implying a weak predictive value. Therefore, the link between body composition and patient prognosis should be interpreted with caution. The clinical indicators frequently identified in the reviewed studies included tumor -related factors (e.g., tumor size and number, the Tumor, Node, Metastasis stage, vascular invasion and the alpha-fetoprotein level), liver function-related factors (such as albumin and bilirubin level, Child-Pugh grade), and a combination of these two (like BCLC stage). These findings were consistent with previous research [9,12]. Except these, treatment related factors have also shown to be closely correlated with the prognosis after TACE treatment, including number of TACE sessions [55], and response to TACE [56]. Furthermore, TACE may combine with other treatment options like tyrosine kinase inhibitors, radiofrequency ablation or immunotherapy, which also exerts significant effects on the treatment outcomes [57]. However, these factors were less evaluated in the reviewed studies. A recent international study with 4621 HCC patients treated with TACE in 11 medical centers, two prediction models (“Pre‐TACE‐Predict” and “Post‐TACE‐Predict”) were built based on tumor-, liver function- and treatment-related factors for survival outcome prediction [58]. Those models showed superior to the existing prediction models, like the hepatoma arterial embolization prognostic (HAP) score [58]. However, until now, there is not any research incorporating body composition factor into a prediction model. Further solid evidence is still required to validate the value of body composition in the prediction of the TACE treatment outcome.

This study has a few limitations. First, the number of studies included in this review was limited. Most studies were performed at a single medical center and only one prospective study was included, and. Besides, the total patient population was also limited, and the median sample size was only 165. All of these may undermine a convincing conclusion drawn from this review. Second, meta-analysis was not carried out on all body composition due to the limited study number and obvious heterogeneity (for instance, only one study on bone mineral density and parameters of body composition changes varied). Third, this study only focused on the TACE procedure, not involving other transcatheter intra-arterial therapies, given that the technique heterogeneity is notable in loco-regional interventional therapies. Generally, transcatheter intra-arterial therapy includes bland TAE, TACE, hepatic arterial infusion chemotherapy, and *trans*-arterial radioembolization (such as the use of Yttrium-90) [59,60]. As the investigation in this field is still at an initial stage, there are only a few studies describing each technique, and an accumulation of studies is required before comparing the combined effect of body composition on prognosis after transcatheter intra-arterial therapies. Four, as lacking information from the 14 original studies, the relationship between chronic liver disease, prognosis, and sarcopenia was unavailable to evaluate. As sarcopenia is prevalent in patients with chronic liver disease, their associations with prognosis after TACE is worthwhile further exploration. Lastly, the different embolic agents used in the TACE technique might also contribute to a potential confounder for clinical outcomes. Five studies included patients undergoing both conventional TACE and drug-eluting bead TACE procedures. However, they did not perform subgroup analysis to estimate the prognostic differences between these two groups, making extraction of the relevant information impossible to extract. Future research is warranted to evaluate whether sarcopenia has a different impact on the prognosis of liver cancer patients receiving conventional TACE and drug-eluting bead TACE.

In conclusion, body composition seems to be an important prognostic factor for poorer clinical outcomes after TACE treatment in patients with HCC. Interventional radiologists should pay more attention to body composition analysis in patients who are scheduled for TACE treatment. Yet, prospective research with a large sample size involving multiple centers is required to confirm these findings.

## Funding

This work was funded by Karolinska Institutet Erik and Edith Fernstrom Foundation (No. 2023–00979) and 10.13039/501100009782Ruth and Richard Julin Foundation (No.2023-00474). The funders did not involve in the study design, and collection, analysis, and interpretation of data and in writing the manuscript.

## Ethics approval and consent to participate

Not applicable as it is a systematic review.

## Consent for publication

Not applicable.

## Availability of data and materials

The original contributions presented in the study are included in the article/supplementary material. Further inquiries can be directed to the corresponding author.

## Informed consent

Not applicable as it is a review.

## CRediT authorship contribution statement

**Anrong Wang:** Writing – original draft, Methodology, Formal analysis. **Junfeng Li:** Writing – original draft, Validation, Methodology, Formal analysis, Data curation. **Changfeng Li:** Validation, Software, Methodology, Investigation. **Hui Zhang:** Validation, Investigation, Formal analysis. **Yingfang Fan:** Writing – review & editing, Supervision, Methodology, Formal analysis. **Kuansheng Ma:** Writing – review & editing, Supervision, Methodology, Formal analysis. **Qiang Wang:** Writing – review & editing, Validation, Supervision, Project administration, Methodology, Funding acquisition, Formal analysis, Conceptualization.

## Declaration of competing interest

The authors declare the following financial interests/personal relationships which may be considered as potential competing interests: Qiang Wang reports financial support was provided by 10.13039/501100004047Karolinska Institutet. If there are other authors, they declare that they have no known competing financial interests or personal relationships that could have appeared to influence the work reported in this paper.
