# Supplementary material for: Impact of body composition on the prognosis of hepatocellular carcinoma patients treated with transarterial chemoembolization: A systematic review and meta-analysis
Source: Heliyon. 2024 Feb 2;10(3):e25237. doi: 10.1016/j.heliyon.2024.e25237 (PMC10862505; doi:10.1016/j.heliyon.2024.e25237)
Supplement: Multimedia component 2 [file mmc2.docx]

Supplemental sTable 2: The literature searching query (as of May 15, 2023).

| **No.** | **Search Query for PubMed** | **Result** |
| --- | --- | --- |
| #1 | "carcinoma, hepatocellular"[MeSH Terms] OR "Liver Neoplasms"[MeSH Terms:noexp] | **179,144** |
| #2 | ((Hepatocellular[Title/Abstract] OR "liver cell"[Title/Abstract] OR "hepatic cell"[Title/Abstract]) AND carcinoma*[Title/Abstract]) OR (hepatocarcinoma*[Title/Abstract] OR hepatoma*[Title/Abstract] OR "liver carcinoma"[Title/Abstract] OR HCC[Title/Abstract]) | **154,335** |
| #3 | **#1 OR #2** | **239,533** |
| #4 | "chemoembolization, therapeutic"[MeSH Terms] | 7,221 |
| #5 | "transcatheter arterial chemoembolization"[Title/Abstract] OR "transarterial chemoembolization"[Title/Abstract] OR "transcatheter hepatic arterial chemoembolization"[Title/Abstract] | 7092 |
| #6 | **#4 OR #5** | 10,024 |
| #7 | “body composition” [MeSH Terms] OR "sarcopenia"[MeSH Terms] OR "osteoporosis"[MeSH Terms] | 132,655 |
| #8 | "body composition"[Title/Abstract] OR "sarcopenia "[Title/Abstract] OR " analytic morphomics "[Title/Abstract] OR "muscle depletion"[Title/Abstract] OR "muscle mass"[Title/Abstract] OR "psoas area"[Title/Abstract] OR "myopenia"[Title/Abstract] OR "core muscle"[Title/Abstract] OR "lean body mass"[Title/Abstract] OR "muscular atrophy"[Title/Abstract] OR "subcutaneous adipose tissue" [Title/Abstract] OR "visceral adipose tissue" [Title/Abstract] OR "subcutaneous fat" [Title/Abstract] OR "visceral fat" [Title/Abstract] OR “bone mineral density”[Title/Abstract] OR “bone mineral content”[ Title/Abstract] OR “osteopenia” [Title/Abstract] OR “osteoporosis” [Title/Abstract] | 222,429 |
| #9 | **#7 OR #8** | 266,767 |
| #10 | **#3 AND #6 AND #9** | 28 |

| **No.** | **Search Query for Web of Science** | **Result** |
| --- | --- | --- |
| #1 | TS = (((Hepatocellular OR "liver cell" OR "hepatic cell") AND carcinoma*) OR hepatocarcinoma* OR hepatoma* OR "liver carcinoma" OR HCC) | **210,653** |
| #2 | TS = ("transcatheter arterial chemoembolization" OR " transarterial chemoembolization" OR " transcatheter hepatic arterial chemoembolization") | 9,540 |
| #3 | TS = (" body composition" OR "analytic morphomics" OR "sarcopenia " OR "muscle depletion" OR "muscle mass" OR "psoas area" OR "myopenia" OR "core muscle" OR "lean body mass" OR "muscular atrophy" OR "subcutaneous adipose tissue" OR "visceral adipose tissue" OR "subcutaneous fat" OR "visceral fat" OR “bone mineral density” OR “bone mineral content” OR “osteopenia” OR “osteoporosis” ) | 293,945 |
| #4 | **#1 AND #2 AND #3** | 40 |

| **No.** | **Search Query for Embase** | **Result** |
| --- | --- | --- |
| #1 | 'liver cell carcinoma'/exp OR 'liver cancer'/de | **326,614** |
| #2 | (((hepatocellular OR 'liver cell' OR 'hepatic cell') NEAR/3 carcinoma*):ab,ti,kw) OR hepatocarcinoma*:ab,ti,kw OR hepatoma*:ab,ti,kw OR 'liver carcinoma':ab,ti,kw OR hcc:ab,ti,kw | **217,978** |
| #3 | #1 OR #2 | **373,731** |
| #4 | "embolization, therapeutic"/exp | 114,164 |
| #5 | "transcatheter arterial chemoembolization":ab,ti OR "transarterial chemoembolization":ab,ti OR "transcatheter hepatic arterial chemoembolization":ab,ti | 11,096 |
| #6 | **#4 OR #5** | 114,742 |
| #7 | "body composition"/exp OR "sarcopenia"/exp OR "osteoporosis "/exp | 290,008 |
| #8 | "body composition":ab,ti OR "analytic morphomics":ab,ti OR "sarcopenia":ab,ti OR "muscle depletion":ab,ti OR "muscle mass":ab,ti OR "psoas area":ab,ti OR "myopenia":ab,ti OR "core muscle":ab,ti OR "lean body mass":ab,ti OR "muscular atrophy":ab,ti OR "subcutaneous adipose tissue":ab,ti OR "visceral adipose tissue":ab,ti OR "subcutaneous fat":ab,ti OR "visceral fat":ab,ti OR “bone mineral density”:ab,ti OR “bone mineral content”:ab,ti OR “osteopenia”:ab,ti OR “osteoporosis”:ab,ti | 312,486 |
| #9 | **#7 OR #8** | 424,165 |
| #10 | **#3 AND #6 AND #10** | 123 |

| **No.** | **Search Query for Cochrane Library** | **Result** |
| --- | --- | --- |
| #1 | Mesh descriptor [Carcinoma, Hepatocellular] OR [Liver Neoplasms] explode all trees | 3,880 |
| #2 | ((hepatocellular OR 'liver cell' OR 'hepatic cell') NEAR/6 carcinoma*):ab,ti,kw OR (hepatocarcinoma* OR hepatoma* OR 'liver carcinoma' OR HCC):ab,ti,kw | 30,355 |
| #3 | #1 OR #2 | 31,563 |
| #4 | MeSH descriptor: [embolization, therapeutic] explode all trees | 1,065 |
| #5 | "transcatheter arterial chemoembolization":ab,ti,kw OR "transarterial chemoembolization":ab,ti,kw OR "transcatheter hepatic arterial  chemoembolization":ab,ti,kw | 1,033 |
| #6 | **#4 OR #5** | 1,842 |
| #7 | Mesh descriptor [Body composition] OR [Sarcopenia] OR [Osteoporosis] explode all trees | 13,422 |
| #8 | "body composition":ti,ab,kw OR "analytic morphomics":ti,ab,kw OR "sarcopenia ":ti,ab,kw OR "muscle depletion":ti,ab,kw OR "muscle mass":ti,ab,kw OR "psoas area":ti,ab,kw OR "myopenia":ti,ab,kw OR "core muscle":ti,ab,kw OR "lean body mass":ti,ab,kw OR "muscular atrophy":ti,ab,kw OR "subcutaneous adipose tissue":ti,ab,kw OR "visceral adipose tissue":ti,ab,kw OR "subcutaneous fat":ti,ab,kw OR "visceral fat":ti,ab,kw OR “bone mineral density”:ti,ab,kw OR “bone mineral content”:ti,ab,kw OR “osteopenia”:ti,ab,kw OR “osteoporosis”:ti,ab,kw | 40,740 |
| #9 | #7 OR #8 | 41,323 |
| #10 | **#3 AND #6 AND #9** | 1 |

Supplemental sTable 3: Chronic hepatitis information and its association with body composition and prognosis.

| Study ID | Chronic hepatitis | Difference of body composition † | Hazards ratio^#^ |
| --- | --- | --- | --- |
| Parikh et al.[23] | HCV:63 (84%)+55 (61%) | NA | NA |
| Cheng et al. [24] | HBV:4(4.7%);  HCV:46 (53.5%); HBV+HCV:9(10.5%) | NA | NA |
| Fujita et al. [25] | HBV:24(13.4%);  HCV:85(47.5%) | HBV/HCV: normal PMI vs low PMI groups (**NS**) | NA |
| Loosen et al. [26] | Unclear | NA | NA |
| Hashida et al. [27] | HBV:12(7.9%);  HCV:105(69.1%) | **Not significant** difference in ΔSMI among the different etiology groups (HBV vs HCV vs Autoimmune hepatitis vs Alcohol vs NASH) | NA |
| Li et al. [28] | HBV:122 (63.5%) | NA | NA |
| Lim et al. [29] | HBV:155 (58.3%);  HCV:60(22.6%) | NA | HCV: 1.80 (95%CI:1.30–2.51) (**significant**, but multivariable Cox analysis not available) |
| Zheng et al. [30] | Unclear | NA | NA |
| Zhang et al. [31] | HBV:194 (85.1%) | HBV: High vs Low SMI groups (**NS**); High vs Low PMI groups (**NS**) | HBV:1.18 (95%CI:0.74–1.86) **(NS)** |
| Muller et al. [32] | Viral hepatitis: 270 (29.7%) | NA | NA |
| Chien et al. [33] | HBV:141(54.2%);  HCV:110(42.3%) | HBV: non-sarcopenia vs sarcopenia groups **(NS)**; HCV: non-sarcopenia vs sarcopenia groups (**NS**) | NA |
| Loosen et al-2[34] | HBV:14.3%;  HCV:27.1% | Median muscular attenuation was **significantly** higher  in HBV or/and HCV group than other etiology group (alcohol, NASH) | NA |
| Bannangkoon et al. [35] | HBV:301 (49.3%);  HCV:135 (22.1%);  HBV+HCV:7 (1.1%) | NA | NA |
| Wang et al. [36] | HBV:247 (67.9%) | NA | NA |

*Note: † in HBV/HCV group vs non-HBV/HCV group unless otherwise noted; # in univariable Cox regression analysis; CI, confidence interval; HBV, hepatitis B virus; HCV, hepatitis C virus; NA, not available; NS, not significant.*
